# *Aedes aegypti* microbiome composition covaries with the density of *Wolbachia* infection

**DOI:** 10.1186/s40168-023-01678-9

**Published:** 2023-11-17

**Authors:** Jane Pascar, Henry Middleton, Steve Dorus

**Affiliations:** Center for Reproductive Evolution, Department of Biology, Syracuse University, Syracuse, NY USA

**Keywords:** Dengue virus, Core microbiome, Vector competency, Mosquito, Pathogen blocking

## Abstract

**Background:**

*Wolbachia* is a widespread bacterial endosymbiont that can inhibit vector competency when stably transinfected into the mosquito, *Aedes aegypti*, a primary vector of the dengue virus (DENV) and other arboviruses. Although a complete mechanistic understanding of pathogen blocking is lacking, it is likely to involve host immunity induction and resource competition between *Wolbachia* and DENV, both of which may be impacted by microbiome composition. The potential impact of *Wolbachia* transinfection on host fitness is also of importance given the widespread release of mosquitos infected with the *Drosophila melanogaster* strain of *Wolbachia* (*w*Mel) in wild populations. Here, population-level genomic data from *Ae. aegypti* was surveyed to establish the relationship between the density of *w*Mel infection and the composition of the host microbiome.

**Results:**

Analysis of genomic data from 172 *Ae. aegypti* females across six populations resulted in an expanded and quantitatively refined, species-level characterization of the bacterial, archaeal, and fungal microbiome. This included 844 species of bacteria across 23 phyla, of which 54 species were found to be ubiquitous microbiome members across these populations. The density of *w*Mel infection was highly variable between individuals and negatively correlated with microbiome diversity. Network analyses revealed *w*Mel as a hub comprised solely of negative interactions with other bacterial species. This contrasted with the large and highly interconnected network of other microbiome species that may represent members of the midgut microbiome community in this population.

**Conclusion:**

Our bioinformatic survey provided a species-level characterization of *Ae. aegypti* microbiome composition and variation. *w*Mel load varied substantially across populations and individuals and, importantly, *w*Mel was a major hub of a negative interactions across the microbiome. These interactions may be an inherent consequence of heightened pathogen blocking in densely infected individuals or, alternatively, may result from antagonistic *Wolbachia*-incompatible bacteria that could impede the efficacy of *w*Mel as a biological control agent in future applications. The relationship between *w*Mel infection variation and the microbiome warrants further investigation in the context of developing *w*Mel as a multivalent control agent against other arboviruses.

Video Abstract

**Supplementary Information:**

The online version contains supplementary material available at 10.1186/s40168-023-01678-9.

## Background

*Aedes* mosquitoes are a primary vector for dengue (DENV) and other arboviruses, including Zika, chikungunya, and yellow fever [1]. Amongst these, DENV transmission poses a particularly severe risk to human health as it is responsible for 50–100 million infections annually across the approximately 125 countries in which it is endemic [2–5]. Above and beyond existing strategies for *Aedes* population control (*e.g.,* insecticides and other chemical or physical management mechanisms) alternative biological approaches can contribute to sustained disease reduction [6]. For example, substantial reductions in DENV transmission have been achieved through the release of mosquitoes stably infected with the bacterial symbiont *Wolbachia* [7–11], which confers resistance to DENV and other arboviruses (commonly referred to as “pathogen blocking”) [12–15].

*Wolbachia pipientis* is a maternally inherited gram-negative endosymbiotic bacterium that is estimated to naturally infect at least two-thirds of arthropod species [16]. *Wolbachia* facilitates its spread within populations through a variety of mechanisms that manipulate host reproduction, including cytoplasmic incompatibility, male feminization, male killing, and the induction of parthenogenesis [17]. The *Wolbachia* strain *w*Mel, which naturally infects *Drosophila melanogaster,* has been developed to stably transinfect *Ae. aegypti*. In this host, *w*Mel induces cytoplasmic incompatibly, whereby uninfected males can produce viable offspring with *w*Mel-infected females but matings between *w*Mel-infected males with uninfected females result in embryonic death [18]. Thus, *w*Mel can spread rapidly and then become stably maintained in populations [19, 20]. However, recent studies have documented fitness reductions in individuals harboring *wMel* that have the potential to slow the spread or reduce the persistence of transinfected individuals [21, 22]. This, in turn, has led to the identification of locally adapted *w*Mel strains with higher fitness that can be strategically deployed to improve biocontrol outcomes [23–25].

Two primary mechanisms are believed to form the basis of *w*Mel induced pathogen blocking: (1) induction of innate host immunity and (2) resource competition between microbes [26]. Pathogen blocking due to *w*Mel-induced immunity is supported by several observations. First, *w*Mel confers a partial antiviral effect against RNA viruses in *D. melanogaster* and viral inhibition in mosquito cell lines [27, 28]. Second, wMel infection in *Ae. aegypti* induces the upregulation of the *Toll* and *Imd* immunity pathways that target pathogens for removal through the production of antimicrobial peptides [29–31]. Third, a mechanistic link between immunity induction and pathogen blocking is further supported by the fact that DENV infection in *Ae. aegypti* triggers a *Toll* pathway-mediated response and that *Wolbachia* density itself decreases during DENV infection [32, 33]. The second mechanism implicated in pathogen blocking relates to competition for host resources [34–36]. As an obligate endosymbiont, *Wolbachia* (much like viruses) relies on a suite of host resources. For example, neither *Wolbachia* nor DENV are able to independently synthesize cholesterol [35]. *w*Mel infection in *Ae. aegypti* increases cholesterol storage in lipid droplets and inhibits viral replication [36]. Release of cholesterol back into the cytosol reverses this inhibition, and pathogen blocking is also reduced in other insects when raised on a high-cholesterol diet [34]. It is also important to note that the strength of pathogen blocking is variable and has been found to positively correlate with variation of *Wolbachia* infection density across strains [37, 38]. For example, the higher-density infections characteristic of *w*MelPop, relative to *w*Mel, is particularly effective in reducing *Ae. aegypti* DENV vectoring capacity across DENV serotypes [39].

The density and distribution of *Wolbachia* infection across tissues may also be dependent on interactions with the *Aedes* microbiome, which in turn may impact the efficacy of *Wolbachia* as a biological control agent. In fact, several commensal microbes, such as species in the genera *Serratia* and *Asaia*, have already been demonstrated to inhibit stable *Wolbachia* transinfection [40–42]. As such, a more wholistic understanding of the tripartite relationships between the *Ae. aegypti* host, *Wolbachia* and the microbiome is therefore a priority. A previous investigation of *w*Mel infection on the *Ae. aegypti* microbiome found no decrease in compositional richness, but did reveal a reduction in a suite of low abundance bacterial taxa [43]. Furthermore, no changes were observed among species with known *w*Mel incompatibilities (*e.g., Serratia* and *Asaia*). It is important to note, however, that this study utilized laboratory-raised *Ae. aegypti* and it is unclear to what extent these results apply to wild populations. Here, we aim to address this uncertainty through the establishment of a refined understanding of *Ae. aegypti* microbiome variation in populations subject to previous releases of *w*Mel infected individuals.

To assess the influence of *w*Mel infection on microbiome composition, we analyzed available short-read genomic DNA sequence data from a previous study of 172 *Ae. aegypti* females reared in standard conditions from eggs collected in the wild from six geographic locations [20]. These locations included four sites with historic releases of *w*Mel-infected females (Bungalow, Cairns North West, Parramatta Park North and Westcourt) and two sites without historic releases (Cairns North East and Parramatta Park South). Our approach (1) achieved microbiome classifications at the species-level for 94.6% of the nearly 21 million reads that mapped to bacterial genomes and greatly expanded upon previous microbiome characterizations in this species, (2) quantified microbiome variation while also identifying core bacterial species at the population-level, (3) identified substantial variation in the density of *w*Mel infection across individuals and (4) revealed that *w*Mel was a central hub of negative interactions within a larger *Ae. aegypti* microbiome interaction network.

## Methods

### *Ae. aegypti* genome sequencing data

Double digest restriction-site associated DNA sequencing data (ddRAD-seq) were utilized from a study of *Ae. aegypti* populations from six geographic locations near Cairns, Australia (NCBI BioProject PRJNA412140) [20]. Releases of *Ae. aegypti* transinfected with the *D. melanogaster* strain of *Wolbachia* (*w*Mel) were conducted in four of these locations: Parramatta Park North and Westcourt (2013 releases); Bungalow and Cairns North West (2014 releases). Schmidt et al. [20] conducted Illumina HiSeq2500 (100 bp reads) paired-end ddRADseq library sequencing of DNA isolated from 172 female mosquitoes reared using standardized conditions from eggs collected in the wild. We have taken advantage of the fact that whole mosquito genomic DNA isolation also captures microbiome DNA. Additional information about the samples can be found in the metadata for NCBI BioProject PRJNA412140.

### Taxonomic classification *Ae. aegypti* microbiome

The Kraken2 pipeline [44] was used to assign taxonomic classifications to all raw ddRAD-seq reads that met standard quality metrics. The Kraken2 search database included all available RefSeq genomes from bacteria (*n* = 21,333), fungi (*n* = 60), protozoa (*n* = 40), viruses (*n* = 10,388), archaea (*n* = 390) (NCBI RefSeq Release Number 200 on August 26, 2020) and the recently reannotated *Ae. aegypti* reference genome (AaegL5 [45]). The database was built with 512 GB of available RAM on 4 threads using the default Kraken2 parameters for minimizer length and spaces, and a conservative k-mer length of 31. Low complexity regions of genomes were masked using the DustMasker program [46]. As expected, the vast majority of reads (93.6% average across 172 individuals; SD = 2.6%) mapped to the *Ae. aegypti* genome. One percent of reads (185,811 reads on average per individual; range: 29,951 – 4,350,613) mapped to bacterial, viral, archaeal, fungal, or protozoan genomes.

### Quantification of *Ae. aegypti* microbiome composition

After taxonomic classification at the species level for bacterial, viral, archaeal, fungal, or protozoan reads, Bracken [47] was used quantify species abundances per individual. The Bracken database was built using default parameters and a read length of 100. Following Bracken quantification, species abundance estimates were standardized to account for variation in the overall sequencing depth per individual. Additionally, we controlled for the number of restriction enzyme cut sites per genome using a script that calculated the total number of *NlaIII* (CATG/GTAC) and *MluCI* (AATT/TTAA) enzyme recognition sites in each reference genome (code is provided in Additional file 1). Standardizations were conducted separately in each major taxonomical group (*e.g.,* bacterial, fungi, etc*.)*. An independent verification of quantitative accuracy was conducted using the original *w*Mel qPCR abundance estimates from Schmidt et al. [20]. For the full set of 172 individuals, abundance estimates were highly reproducible (*r* = 0.87; *p* < 0.0001). Additionally, we detected *w*Mel, albeit at low average abundance levels, in individuals that were previously deemed to be uninfected by qPCR. After removing these datapoints, the quantitative reproducibility between studies increased (*r* = 0.92; *p* < 0.0001).

For subsequent analyses, rare species were removed from the dataset if they (1) did not constitute > 1% of the total bacterial microbiome in any individual and (2) were present in fewer than 25% of individuals. We note that our analysis was based on DNA isolated from whole mosquitos. It therefore reflects the sum of DNA from microbial populations per individual and it does not provide direct information about tissue-specific microbiome composition.

### Statistical analysis of bacterial microbiome composition

Microbiome complexity was assessed in R [48] using two alpha diversity metrics: observed species richness and Shannon’s diversity index [49]. A linear model was used to assess relationships between alpha diversity measures and total *w*Mel abundance per individual. An analysis of variance (ANOVA) and Tukey’s Post Hoc test was used to compare the density of *w*Mel across sites with historical releases. Co-occurrence and mutually-exclusive interactions between bacterial species were assessed using Pearson’s correlations as implemented by CoNet [50] in Cytoscape [51] on a standardized bacterial species abundance matrix. The matrix was standardized using the Wisconsin function from the R package vegan (version 2.5–7 [52]). Statistical significance of each pairwise comparison was determined using 1000 bootstrap replications and Benjamini–Hochberg multiple testing correction. The network was visualized using the Cytoscape GUI software [51]. Additional analysis of network composition was based on delineating two subnetworks. The first (hereafter referred to as the *“w*Mel subnetwork”) includes those species with a significant edge interaction directly with *w*Mel and the second (hereafter referred to as the “non-*w*Mel subnetwork”) as those species without a direct edge interaction with *w*Mel.

Principal Coordinate Analysis (PCoA) was then used to further characterize bacterial microbiome variation. A distance matrix was calculated by applying the Bray–Curtis dissimilarity index [53] implemented by the vegdist function from the R package vegan (version 2.5–7 [52]) to the same standardized matrix used the CoNet analysis (see above). The PCoA was performed using the pcoa function from the R package ape (version 5.5 [54]). Axes of variation were further investigated using linear models to assess the relationship between Principal Coordinate (PC) axis loading values and standardized species abundance estimates.

## Results

### *Ae. aegypti* microbiome composition

**Fig. 1:**
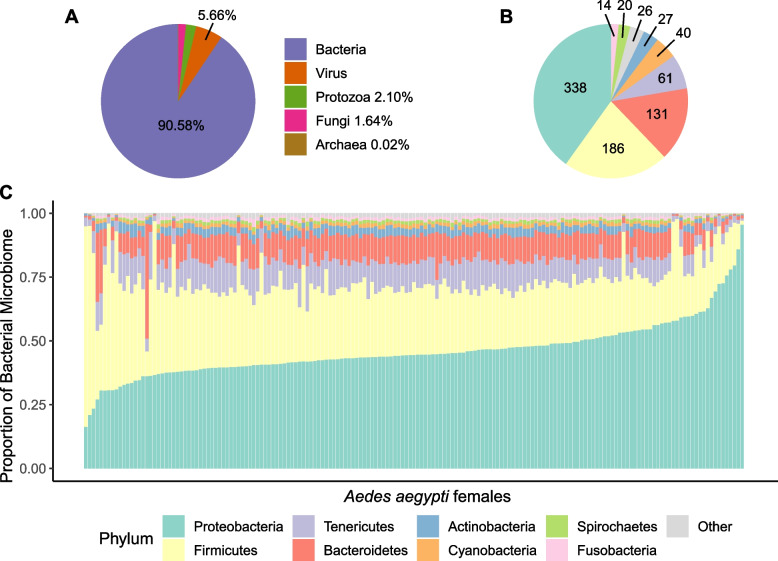
Population-level *Ae. Aegypti* microbiome characterization*.* **A** Taxonomical distribution of reads mapping to non-*Ae. Aegypti* genomes. **B** Taxonomical distribution of the major phyla in the bacterial microbiome. **C** Bacterial microbiome composition by phyla for all 172 individuals. The eight most abundant phyla are shown and the remaining are grouped as ‘Other’

The overarching goal of this study was to bioinformatically characterize microbiome variation, including that of *w*Mel, in a wild population of *Ae. aegypti*. To this end, DNA-seq reads from 172 *Ae. aegypti* females were mapped to a database containing the *Ae. aegypti* genome and the full set of RefSeq genomes from bacteria, fungi, viruses, archaea and protozoans (27,435 genomes in total). Data from each individual resulted in an average of 18.7 million mapped reads (SD = 220,395), of which the vast majority mapped to the *Ae. aegypti* reference genome (93.6%; SD = 2.6%). On average, 5.4% (SD = 1.1%) of reads remained unclassified because they were either low quality reads or derived from species not included in our database. The remaining reads (185,811 reads per individual on average) mapped to bacterial, viral, archaeal, fungal, or protozoan genomes. Of these, the vast majority (90.6%; SD = 5.9%) were derived from bacteria (Fig. 1A). Fungi, protozoa, and archaea were consistently identified at low abundances, while viral load was more variable. As expected, given that our analysis was based on DNA sequencing data, 99.8% of all viral reads identified from bacteriophage (*i.e.,* DNA viruses) and the observed viral load variation was due to the high abundance of *Escherichia* bacteriophages in a limited number of individuals. Full information regarding fungal, viral, archaeal, and protozoan identifications can be found in Additional file 2.

### Bacterial microbiome composition

After removing low abundance and rare species, 843 bacterial species remained from 23 phyla and 396 genera (Additional file 3; note that *w*Mel is not included in the following analysis of microbiome composition). These results represent a substantial increase in the size and taxonomical specificity of the characterized *Ae. aegypti* microbiome. Eight phyla comprised > 99.0% of total bacterial composition (Fig. 1B). Consistent with previous studies, all of which relied on 16S rRNA sequencing [43, 55–59], Proteobacteria (49.0% of bacterial reads) was identified as the predominant phylum. Firmicutes (36.6%), Tenericutes (5.9%), Bacteroidetes (4.7%), Actinobacteria (1.5%), Cyanobacteria (0.6%), Spirochaetes (0.5%), and Fusobacteria (0.5%) were also identified as substantive contributors to the microbiome (Fig. 1C). At the genus level, our analysis confirmed the presence of several previously reported groups, including (1) *Acinetobacter* and *Stenotrophomonas*, which have been identified in reproductive tissues, (2) *Bacillus, Chryseobacterium, Enterobacter*, *Klebsiella and Serratia,* which are commonly identified in the midgut, (3) *Burkholderia*, which has been identified specifically in the salivary glands and (4) a suite of six genera with more complex spatial distributions across multiple tissues [43, 55–58, 60–63]. We also identified several novel species within the *Mycoplasma, Salmonella,* and *Mannheimia* genera that were either ubiquitous among individuals or highly abundant. Finally, several taxa (*e.g.,* species in the *Asaia*, *Spiroplasma* and *Serratia* genera) have been demonstrated to negatively influence *Wolbachia* density or transmission [40, 41, 64]. Our analysis did not identify any species in the genus *Asaia*, consistent with Audsley et al*.* [65], but did identify 17 species in the genera *Spiroplasma* and three in the genera *Serratia*. Among these species, a negative abundance relationship was observed between *w*Mel and *S. marcescens* (see below).

### Towards a core *Ae. aegypti* bacterial microbiome

**Fig. 2:**
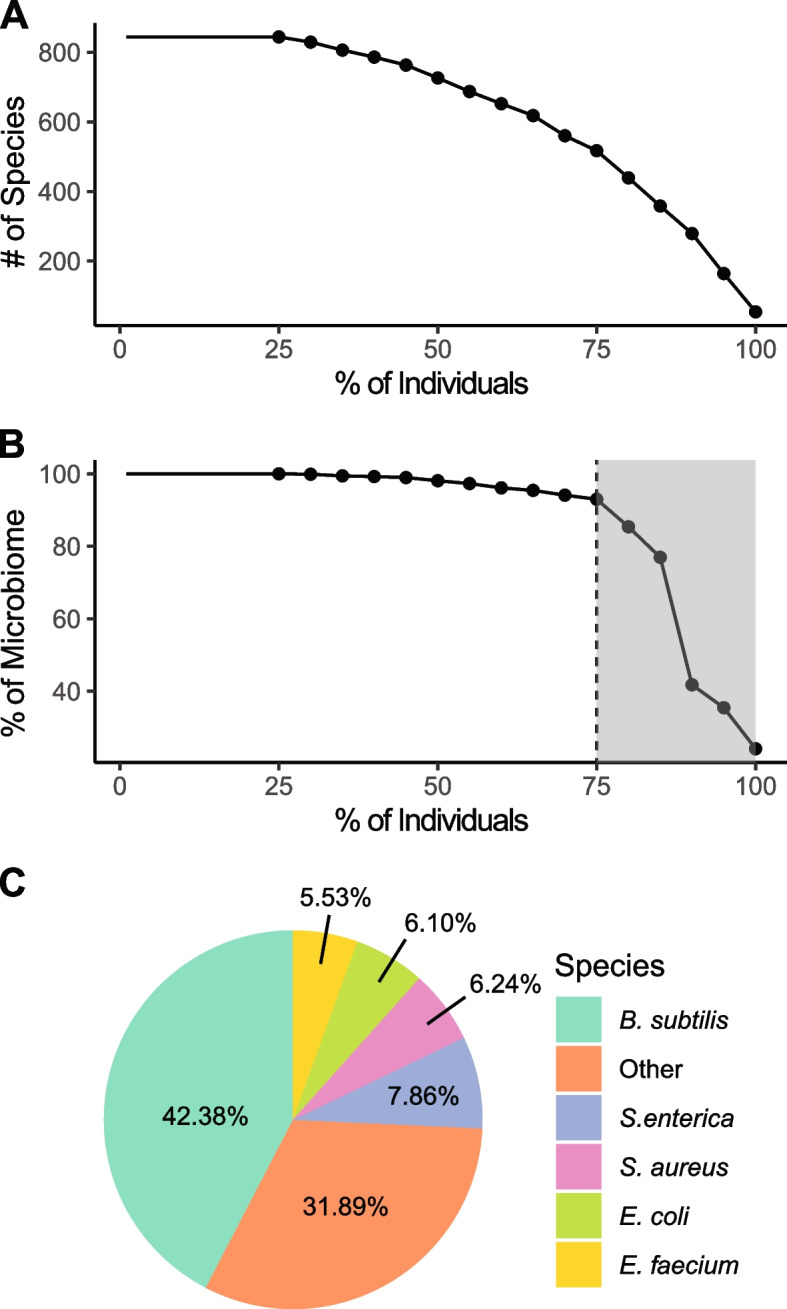
*Ae. Aegypti core microbiome*. **A** Number of bacterial species is plotted in relation to the proportion of individuals in which each species was identified. **B** Percent of microbiome composition is plotted in relation to the proportion of individuals in which each species was identified. Those species present in 75% or more individuals comprised 93% of total microbiome composition. Fifty-four species were identified in all 172 individuals. **C** Contribution of the 54 ubiquitously present microbiome species to core microbiome composition. Species that do not constitute > 5% of the core microbiome have been grouped as ‘Other.’

Our analysis provided a unique opportunity to leverage intraspecific variation to assess the community composition of the core *Ae. aegypti* microbiome at a population level. First, we investigated core microbiome composition as a function of species presence across the population. This analysis revealed that 164 species were present in 95% of individuals and 54 species (hereafter referred to as the “core microbiome”) were present consistently across the entire population (Fig. 2A; Additional file 4). Species present in large numbers of individuals also constituted a high proportion of total microbiome composition (Fig. 2B). Specifically, the 517 bacterial species identified in at least 75% of individuals comprised 93.0% of the total bacterial microbiome. The remaining 7.0% of the microbiome was a combination of 326 additional species that were of far lower abundance on average. Among the 54 species comprising the core microbiome, five species constituted more than 5% of the core microbiome on average (Fig. 2C). *Bacillus subtilis* (42.4%) had the highest average abundance by far, followed by *Salmonella enterica* (7.9%), *Staphylococcus aureus* (6.2%), *Escherichia coli* (6.1%), and *Enterococcus faecium* (5.5%). The high abundance of each of these species was likely due to their contribution to the midgut microbiome (*Bacillus subtilis*, *Salmonella sp., Staphylococcus sp., Escherichia sp.,* and *Enterococcus sp.* have all been previously identified in the *Ae. aegypti* midgut microbiome [60, 63, 66–69]). Although it is noteworthy that several of these species are mammalian pathogens and we cannot rule out possible contamination. Species in the genera *Acinetobacter, Clostridium*, *Cupriavidus*, *Klebsiella, Serratia* were also identified as members of the core microbiome. These genera have all been previously identified in mosquitoes but were not associated with a particular tissue [43, 65, 70, 71]. Unexpectedly, 62.3% (or 34 species) of the core microbiome was comprised of species previously unidentified in the *Ae. aegypti* microbiome (Additional file 4).

### Population distribution of *w*Mel

**Fig. 3:**
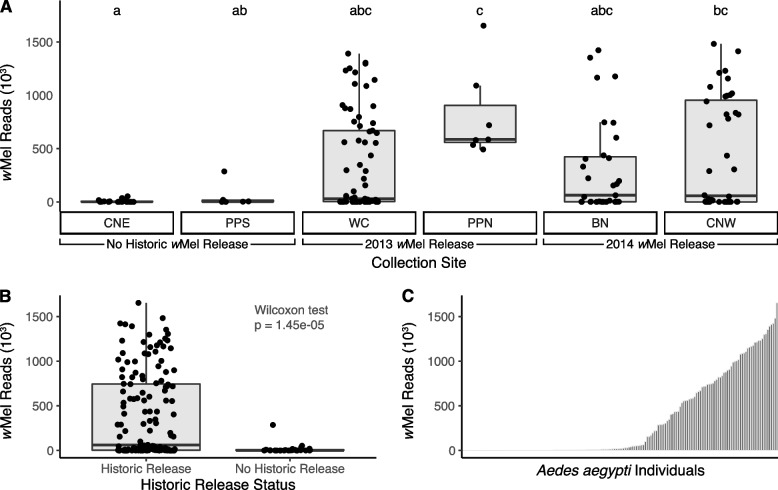
*w*Mel variation across population and individuals. **A** *w*Mel abundance (normalized read count) per individual for each collection site (status and date of previous *w*Mel release is indicated): Bungalow (BN), Cairns North West (CNW), Cairns North East (CNE), Parramatta Park North (PPN), Parramatta Park South (PPS) and Westcourt (WC) [20]. ANOVA revealed significant variation between the *w*Mel abundance across sites, *p* = 0.0006. A post hoc Tukey test revealed that *w*Mel in CNE was significantly less than those from PPN (*p* = 0.005) and CNW (*p* = 0.001) and in PPS was significantly less than those from PPN (*p* = 0.049). **B** *w*Mel abundance (normalized read count) per individual comparing sites with and without historic releases. Wilcoxon test revealed significantly higher *wMel* abundance in sites with historic releases (*p* = 1.45e-05). **C** Individuals from all populations ranked by total *w*Mel abundance

Historic releases of *w*Mel-infected females were conducted in four of the six populations analyzed here, within which stable *w*Mel inheritance has already been documented [20]. Based on recent observations from controlled releases, which revealed very limited geographic dispersal of transinfected individuals [8], we predicted a substantively higher prevalence of *w*Mel in locations with historic releases. This was confirmed by an ANOVA that showed significant variation between the *w*Mel across all sites (*p* = 0.0006; Fig. 3A), including significantly higher abundance levels in the populations with historic releases relative to those without historic releases (*p* = 1.45 × 10^–5^; Fig. 3B). Nevertheless, a substantial amount of intraspecific variation in *w*Mel abundance was also observed in populations with historic releases. Overall, average *w*Mel abundance across all populations was 460,458 (coefficient of variance = 1.05) and ranking individuals by *wMel* abundance revealed a largely continuous gradient of infection density with the highest density infections exceeding 1.5 million reads (Fig. 3C). Given that the strength of pathogen blocking is dependent on the density of *Wolbachia* infection [38], we next sought to assess the relationship between such variation and bacterial microbiome composition.

### wMel was a hub of mutually exclusive relationships with other microbiome species

**Fig. 4:**
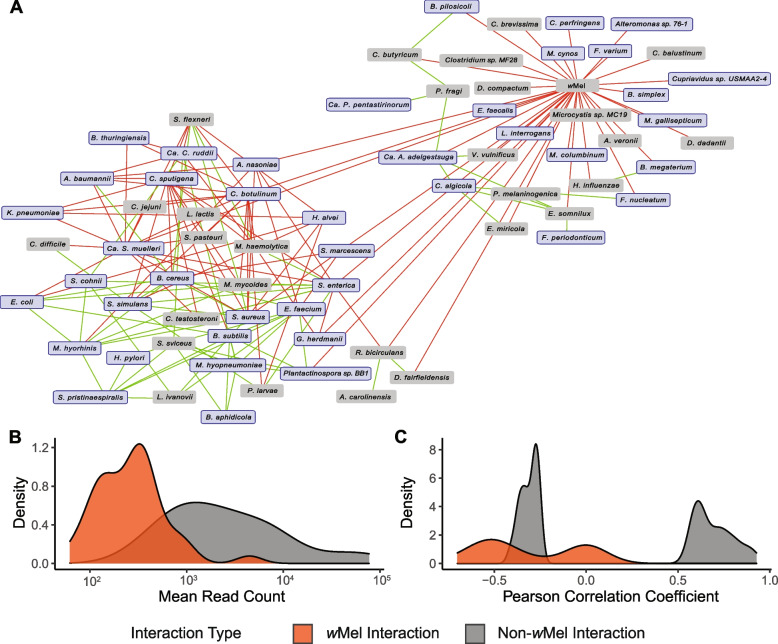
*w*Mel forms a negative interaction hub within the *Ae. Aegypti* microbiome network. **A** Microbiome interaction network analysis using Wisconsin normalized read counts resulted in a network of 71 species and 159 edges. Red edges represent mutual exclusion interactions (significant negative Pearson’s correlation coefficient) and green edges represent co-occurrence interactions (significant positive Pearson’s correlation coefficient). Core microbiome species are highlighted in purple. *w*Mel was involved exclusively in negative pairwise interactions with 35 species. **B** Density plot displaying the strength of interactions edges with *w*Mel (orange) and the remaining edges in the network not involving *w*Mel (grey). **C** Density plot displaying the mean read count for species included in the network. Species with mutually exclusive interactions with *w*Mel are shown in orange and have a significantly lower average read count (t = 2.43; *p*-value = 0.02) than all other species in the network (grey)

*w*Mel abundance did not correlate with either total bacterial abundance (p = 0.66) or observed species richness (*p* = 0.89). However, there was a significant inverse relationship between *w*Mel abundance and Shannon’s Index (*p* = 0.02). Thus, as *w*Mel abundance increased, the relative abundance of other bacterial species in the microbiome became less uniform. A previous investigation of *w*Mel-infected *Ae. aegypti* revealed a reduction in several low abundance genera across the microbiome, but did not report information about *w*Mel infection variation [43]. To specifically explore the effect of *w*Mel abundance, we constructed an interaction network to assess patterns of co-occurrence and mutual exclusion within the microbiome. This approach revealed a network of 71 species, which was significantly enriched for core microbiome species (obs. = 42; exp. = 4.6; χ^2^ = 355.7; p = 2.4 × 10^–79^) and comprised two predominant subnetworks (Fig. 4a; Additional file 5). The first subnetwork (hereafter referred to as the “*w*Mel subnetwork”) comprised 35 mutually-exclusive bacterial relationships specifically with *wMel* and had a low relative level of connectivity (average of 1.5 edges per node). The second, larger subnetwork (hereafter referred to as the “non-*w*Mel subnetwork”) comprised 56 species and was notable for a higher average level of connectivity (2.2 edges per node), and both co-occurrence (*n* = 66) and mutually exclusive (*n* = 58) interaction edges. Three additional characteristics further distinguished these subnetworks. First, the *w*Mel subnetwork comprised species identified at significantly lower abundances than those in the non-*w*Mel subnetwork (Fig. 4B) despite the proportion of core microbiome species being indistinguishable between the two (χ^2^ = 0.45; *p* = 0.50). Second, interaction edges in the *w*Mel subnetwork were significantly weaker in their absolute magnitude than those in the larger network (Fig. 4C; *p* = 8.38 × 10^–6^). Third, the larger network included *B. subtilis*, a confirmed *Ae. aegypti* midgut microbe and the highest abundance species in this study, as well as numerous other high abundance taxa previously identified in the *Ae. aegypti* midgut (*e.g., B. cereus/thuringiensis, E. faecium, E. coli, S. aureus/simulans/cohnii, S. enterica*). Thus, the larger subnetwork was more robustly interconnected and contained a diversity of abundant taxa from the midgut microbiome. In contrast, *w*Mel serves as a hub of negative pairwise relationships with relatively low abundance species, even though many of these are part of the core microbiome. We propose that this structure may derive from more direct interactions between *w*Mel and members of the reproductive microbiome, given the higher abundance of wMel in reproductive tissues relative to somatic tissues [72].

### Microbiome independent and dependent components of *w*Mel variation

**Fig. 5:**
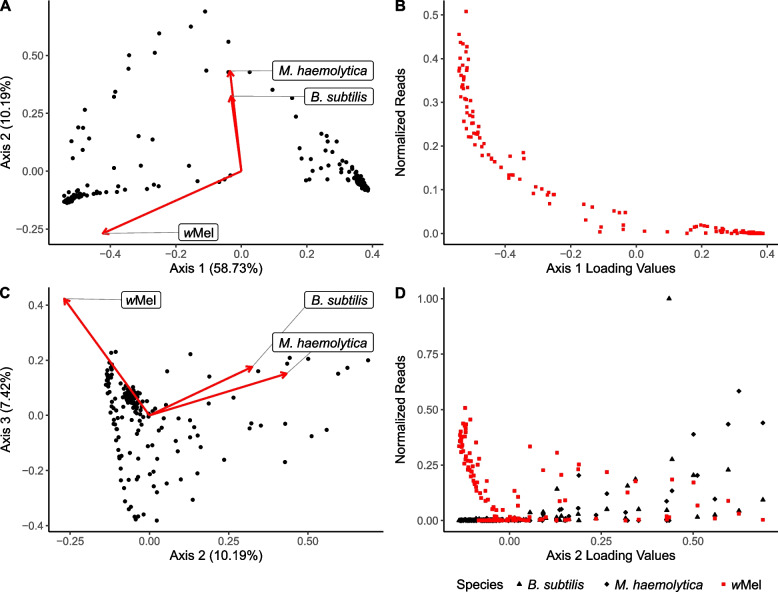
Covariance between microbiome composition and *w*Mel abundance. **A** PCoA analysis of microbiome variation identified a primary axis of variation (PC1: 58.73%) that corresponded with the eigenvector for *w*Mel which was the largest observed vector. Other large eigenvectors representing highly abundant taxa were largely orthogonal to Axis 1. **B** Axis 1 loadings were significantly correlated with *w*Mel abundance across individuals but not other microbiome species. **C** PCoA analysis of microbiome variation identified a secondary axis of variation (PC2: 10.19%) which corresponded with the eigenvectors for *B. subtilis, M. haemolytica*, and to a lesser extent *w*Mel. **D** Axis 2 loadings were significantly correlated with *B. subtilis* and *M. haemolytica* abundance and anticorrelated with *w*Mel abundance across individuals

To assess overall patterns of variation within the microbiome, we utilized a Principal Coordinate Analysis and observed three main axes that captured 76.34% of the total variation (Fig. 5). The first axis (58.73% of variation) included *w*Mel as the eigenvector with the largest magnitude (Fig. 5A). The contribution of *w*Mel variation to this axis was supported by the fact that Axis 1 loadings exhibited a significant correlation with *w*Mel abundance across individuals (*r* = -0.90; t = -26.78, *p* = 4.60 × 10^–63^; Fig. 5B). Thus, Axis 1 largely captures variation in *w*Mel and this variation was largely orthogonal to the other main eigenvectors. However, we note that, of the 10 species with the highest positive loadings on Axis 1 (*i.e.,* those with loadings opposite to *w*Mel), five participate in direct negative interactions with *w*Mel in the *w*Mel subnetwork (Fig. 4A). Axis 2 also identified *w*Mel as a large negative eigenvector but, in contrast to Axis 1, it included two species with large positive eigenvectors (*i.e., M. haemolytica* and *B. subtilis*; Fig. 5C). The relationship with these species was further supported by a significant positive correlation between Axis 2 loadings and the abundance of *w*Mel (*r* = -0.24; t = -3.21, *p* = 0.0016), and a significant negative correlation with *M. haemolytica* (*r* = 0.72; t = 13.49, *p* < 2.2 × 10^–16^) and *B. subtilis* (*r* = 0.48; t = 7.08, *p* = 3.61 × 10^–11^; Fig. 5D). Notably, both species were present in our network analysis (Fig. 4A) and their interaction connections to *w*Mel were consistent with the identified PCoA relationships. In summary, Axis 1 captured *w*Mel variation and its weak, mutually-exclusive interaction with a suite of species identified in the *w*Mel network, and Axis 2 captured stronger, but indirect, *w*Mel covariation with two of the most abundant species in the microbiome, including the midgut microbe *B. subtilis*. Lastly, we explored whether microbiome composition varied by collection site by PCoA and observed limited evidence of clustering above and beyond the variation between sites with and without releases associated with *w*Mel (Additional file 6).

## Discussion

The release of *Ae. aegypti* mosquitoes transinfected with the *w*Mel strain of *Wolbachia* has proven to be an effective strategy to limit the spread of DENV [12, 73]. However, less is known about the long-term dynamics of infection within populations and how these dynamics might influence the multivalent utility of *w*Mel more broadly as a biological control agent for arboviruses. Given the mechanisms responsible for pathogen blocking (*i.e.,* immune priming and resource competition), it is reasonable to assume that *w*Mel infection stability and density may be dependent on interactions with the remainder of the microbiome. Here, we explore this possibility through a population-level *Ae. aegypti* microbiome survey using a bioinformatic approach that leverages available genomic sequencing data. In addition to achieving a species-level *Ae. aegypti* microbiome characterization from a wild population, our analyses revealed substantial intraspecific variation in *w*Mel density that covaried with microbiome composition. This variation is of potential relevance to the efficacy of pathogen blocking and the implementation of *Wolbachia*-based pathogen control strategies against DENV and other arboviruses.

### Towards an *Ae. aegypti* population-level core microbiome

Interactions between bacterial components of the microbiome and their mosquito host impact important aspects of fitness, including fertility, longevity, and immunity [14, 40, 59]. As such, the identification of a population-level, core microbiome – those bacterial species present across many or all individuals – has the potential to provide unique insights into taxa that support host development and function [74]. Importantly, and unlike previous studies that have relied on laboratory populations [56, 62, 75, 76] or utilized 16S sequencing [56, 57, 65, 71, 75] on wild populations, our survey provides a unique species-level perspective into microbiome structure in an *Ae. aegypti* population. Our analysis was also successful in identifying a relatively large repertoire of core microbiome species. Whereas functional analyses will ultimately be required for a refined understanding of core microbiome-host dynamics [77], several observations are worthy of discussion.

The 54 bacterial species ubiquitously present across all individuals comprised only 24% of the total microbiome. However, when the criteria for inclusion was relaxed (to presence in ≥ 75% of individuals), this group of common species comprised 93% of the total microbiome. As such, the vast majority of the microbiome is comprised of common species at the population level. This observation cannot be accounted for by a possible identification bias towards highly abundant species. Whereas many core species were indeed highly abundant, such as *B. subtilis* that comprises 11.2% of the microbiome on average, the abundance of core species varied by over 1,100-fold (Fig. 4C). Thus, many core species were quite low in relative abundance. This variation is almost certainly explained, at least in part, by the absolute size of tissue-specific microbiomes. We therefore predict that the low average abundance of species in the *w*Mel subnetwork is likely due to their restricted presence in the reproductive microbiome. Similarly, the high average abundance of species in the non-*w*Mel subnetwork, which includes *B. subtilis*, suggests that they may be representatives of a larger midgut microbiome. Overall, the significant enrichment of core microbiome species (of both high and low abundance) in our network analysis supports the presence of concerted functional assemblages within the microbial community. Furthermore, the topology of the network, including the presence of delineated subnetworks, suggests compartmentalization between tissues that may inform tissue-specific microbiome functionality. Some of core species identified here have also been identified in laboratory colonies derived from widespread global populations (*e.g.*, Brazil, Grenada, and India [62, 68, 69, 78]). Surveys across addition natural and lab populations will further refine our understanding of the functional core microbiome and, additionally, the process by which natural microbiomes experience compositional shifts or diversity-decay when reared in laboratory settings.

### Variation in *w*Mel infection density and the microbiome

As a reproductive endosymbiont, *Wolbachia* was conventionally believed to be concentrated within the host’s reproductive tissues. However, it is now well-established that *Wolbachia* can be present at variable levels across many non-reproductive tissues [13, 37]. The density and spatiotemporal distribution of *Wolbachia* varies among host species and strains of *Wolbachia* [37, 79]. However, there is limited information about the spatiotemporal dynamics of *w*Mel in wild *Ae. aegypti* populations despite its relevance to pathogen blocking [38]. Despite *w*Mel being stably established in the populations surveyed here [20], we observed a high level of variation in infection density across individuals and a robust covariation between *w*Mel abundance and specific sets of species with the microbiome. Several facets of these relationships are worth highlighting. First, we observed a significant negative relationship between *w*Mel density and Shannon’s Index, indicating a decrease in the compositional evenness in the microbiome. Previous studies comparing alpha diversity metrics in *Wolbachia*-infected hosts have reported contradicting results [43, 80–82]. Our results, based on a large survey of individuals, suggest that the impact of *w*Mel on the *Ae. aegypti* microbiome is more nuanced than a simple reduction in species richness or load, as previously suggested [43, 80, 81]. Second, *w*Mel served as a hub of consistently mutually-exclusive, but weak, interactions and these interactions were generally with lower abundance species. This pattern is consistent with results reported by Audsley et al*.* [65]. Based on the composition of the subnetworks revealed in our analysis, we speculate that this pattern may reflect a suite of direct interactions with other members of the reproductive microbiome. It is also important to emphasize that, although these species were generally low in abundance, they were significantly enriched for members of the core microbiome. Third, our PCoA analysis revealed robust covariation between *w*Mel and several of the most abundant species in the microbiome, including the midgut microbe *B. subtilis*. Given the importance of the midgut microbiome on immune system function [83], the effect of *w*Mel (either direct or indirect) on the midgut microbiome is worthy of further investigation.

## Conclusion

We demonstrate the underappreciated value of existing whole-organism DNA-seq data in relation to microbiome characterization. Our analysis achieved a high coverage, species-level characterization of the *Ae. aegypti* microbiome, including the delineation of a population-level, core microbiome. Despite the absence of information about tissue-specific microbiome composition, network analyses revealed *w*Mel as a hub of interactions with species likely to be of the reproductive microbiome and a small set of robust, indirect interactions with likely members of the midgut microbiome. Intraspecific variation in *w*Mel infection density, in conjunction with diverse interactions with the native microbiome, may have the potential to impact vector competency. The specific interactions identified here could be leveraged to potentially enhance *w*Mel infection density and identify species that may be antagonistic or incompatible with *Wolbachi*a or third-party players that mediate pathways underlying pathogen blocking.

### Supplementary Information

**Additional file 1.** Script used for taxonomic identification, calculating number of enzyme restriction sites and normalization.

**Additional file 2.** Kraken2 and Bracken species identifications and average abundance for fungi, viruses, archaea, and protozoa.

**Additional file 3.** Kraken2 and Bracken species identifications for the 844 bacterial species (representing 23 phyla and 379 genera) meeting at least one of these conservative inclusion criteria: (1) > 1% of the total bacterial microbiome in at least one individual and (2) present in at least 25% of individuals.

**Additional file 4.** Core microbiome of 164 bacterial species which were present in 95% or more of individuals across the entire population surveyed. Average percent contribution to microbiome composition and presence in the strict core microbiome present in all individuals is also provided.

**Additional file 5.** CoNet network characteristics are provided, including the 159 significant edge interactions between all 71 species and the strength of each interaction (Pearson’s Correlation Coefficient).

**Additional file 6.** **Figure S5.** PCoA of microbiome variation indicating collection site and historic release status. PCoA results including *w*Mel are shown in Panel A and B. Individuals from sites with historic releases almost exclusively have positive loadings on Axis 1and negative loadings on Axis 3. PCoA results excluding *w*Mel are shown in Panel C and D. Limited clustering by collection site or historic release status was observed.

## Data Availability

All data generated or analyzed during this study are included in this published article and its supplementary information files.

## References

[1] Powell JR (2018). Mosquito-borne human viral diseases: why aedes aegypti?. Am J Trop Med Hyg.

[2] Murray NEA, Quam MB, Wilder-Smith A (2013). Epidemiology of dengue: Past, present and future prospects. Clin Epidemiol.

[3] World Health Organization (WHO), Special Programme for Research and Tropical Diseases (TDR). Dengue: Guidelines for diagnosis, treatment, prevention and control. In: Dengue: Guidelines for diagnosis, treatment, prevention and control: new edition. World Health Organization; 2009.

[4] Stanaway JD, Shepard DS, Undurraga EA, Halasa YA, Coffeng LE, Brady OJ (2016). The global burden of dengue: an analysis from the Global Burden of Disease Study 2013. Lancet Infect Dis.

[5] Cattarino L, Rodriguez-Barraquer I, Imai N, Cummings DAT, Ferguson NM. Mapping global variation in dengue transmission intensity. Sci Transl Med. 2020;12. 10.1126/scitranslmed.aax4144 31996463

[6] Achee NL, Gould F, Perkins TA, Reiner RC, Morrison AC, Ritchie SA (2015). A critical assessment of vector control for dengue prevention. PLoS Negl Trop Dis.

[7] Anders KL, Indriani C, Tantowijoyo W, Rancès E, Andari B, Prabowo E, et al. Reduced dengue incidence following deployments of Wolbachia-infected Aedes aegypti in Yogyakarta, Indonesia: a quasi-experimental trial using controlled interrupted time series analysis. Gates Open Res. 2020;4. 10.12688/gatesopenres.13122.1 PMC7403856 32803130

[8] Utarini A, Indriani C, Ahmad RA, Tantowijoyo W, Arguni E, Ansari MR (2021). Efficacy of Wolbachia-infected mosquito deployments for the control of dengue. N Engl J Med.

[9] Nazni WA, Hoffmann AA, NoorAfizah A, Cheong YL, Mancini MV, Golding N (2019). Establishment of Wolbachia strain wAlbB in Malaysian populations of Aedes aegypti for dengue control. Curr Biol.

[10] O’Neill SL, Ryan PA, Turley AP, Wilson G, Retzki K, Iturbe-Ormaetxe I, et al. Scaled deployment of Wolbachia to protect the community from dengue and other aedes transmitted arboviruses. Gates Open Res. 2018;2. 10.12688/gatesopenres.12844.2 PMC6305154 30596205

[11] Ryan PA, Turley AP, Wilson G, Hurst TP, Retzki K, Brown-Kenyon J (2020). Establishment of wMel Wolbachia in Aedes aegypti mosquitoes and reduction of local dengue transmission in Cairns and surrounding locations in northern Queensland Australia. Gates Open Res.

[12] Walker T, Johnson PH, Moreira LA, Iturbe-Ormaetxe I, Frentiu FD, McMeniman CJ (2011). The wMel Wolbachia strain blocks dengue and invades caged Aedes aegypti populations. Nature.

[13] Moreira LA, Iturbe-Ormaetxe I, Jeffery JA, Lu G, Pyke AT, Hedges LM (2009). A Wolbachia symbiont in Aedes aegypti limits infection with dengue, Chikungunya, and Plasmodium. Cell.

[14] McMeniman CJ, Lane RV, Cass BN, Fong AWC, Sidhu M, Wang YF, et al. Stable introduction of a life-shortening Wolbachia infection into the mosquito Aedes aegypti. Science (80- ). 2009;323:141–4. 10.1126/science.1165326 19119237

[15] Flores HA, de Bruyne JT, O’Donnell TB, Nhu VT, Giang NT, Trang HTX (2020). Multiple Wolbachia strains provide comparative levels of protection against dengue virus infection in Aedes aegypti. PLoS Pathog.

[16] Hilgenboecker K, Hammerstein P, Schlattmann P, Telschow A, Werren JH (2008). How many species are infected with Wolbachia? - A statistical analysis of current data. FEMS Microbiol Lett.

[17] Correa CC, Ballard JWO (2016). Wolbachia associations with insects: winning or losing against a master manipulator. Front Ecol Evol.

[18] Turelli M, Hoffmann AA (1991). Rapid spread of an inherited incompatibility factor in California Drosophila. Nature.

[19] Hoffmann AA, Montgomery BL, Popovici J, Iturbe-Ormaetxe I, Johnson PH, Muzzi F (2011). Successful establishment of Wolbachia in Aedes populations to suppress dengue transmission. Nature.

[20] Schmidt TL, Filipović I, Hoffmann AA, Rašić G (2018). Fine-scale landscape genomics helps explain the slow spatial spread of Wolbachia through the Aedes aegypti population in Cairns, Australia. Heredity (Edinb).

[21] Ross PA, Turelli M, Hoffmann AA (2019). Evolutionary ecology of Wolbachia releases for disease control. Annu Rev Genet.

[22] Ross PA, Axford JK, Callahan AG, Richardson KM, Hoffmann AA (2020). Persistent deleterious effects of a deleterious Wolbachia infection. PLoS Negl Trop Dis.

[23] Hague MTJ, Shropshire JD, Caldwell CN, Statz JP, Stanek KA, Conner WR (2022). Temperature effects on cellular host-microbe interactions explain continent-wide endosymbiont prevalence. Curr Biol.

[24] Gu X, Ross PA, Rodriguez-Andres J, Robinson KL, Yang Q, Lau MJ (2022). A wMel Wolbachia variant in Aedes aegypti from field-collected Drosophila melanogaster with increased phenotypic stability under heat stress. Environ Microbiol.

[25] Ross PA, Hoffmann AA (2022). Fitness costs of Wolbachia shift in locally-adapted Aedes aegypti mosquitoes. Environ Microbiol.

[26] Caragata EP, Dutra HLC, Moreira LA (2016). Exploiting intimate relationships: controlling mosquito-transmitted disease with Wolbachia. Trends Parasitol.

[27] Hedges LM, Brownlie JC, O’Neill SL, Johnson KN. Wolbachia and virus protection in insects. Science (80- ). 2008;322:702–702. 10.1126/science.1162418 18974344

[28] Bhattacharya T, Newton ILG, Hardy RW (2020). Viral RNA is a target for Wolbachia-mediated pathogen blocking. PLOS Pathog.

[29] Rancès E, Ye YH, Woolfit M, McGraw EA, O’Neill SL (2012). The relative importance of innate immune priming in Wolbachia-mediated dengue interference. PLoS Pathog.

[30] Pan X, Thiem S, Xi Z. Wolbachia-mediated immunity induction in mosquito vectors. In: Arthropod vector: controller of disease transmission. Elsevier Inc.; 2017. p. 35–58.

[31] Pan X, Zhou G, Wu J, Bian G, Lu P, Raikhel AS (2012). Wolbachia induces reactive oxygen species (ROS)-dependent activation of the Toll pathway to control dengue virus in the mosquito Aedes aegypti. Proc Natl Acad Sci U S A.

[32] Ye YH, Woolfit M, Rancès E, O’Neill SL, McGraw EA (2013). Wolbachia-Associated bacterial protection in the mosquito Aedes aegypti. PLoS Negl Trop Dis.

[33] Xi Z, Ramirez JL, Dimopoulos G (2008). The Aedes aegypti toll pathway controls dengue virus infection. PLoS Pathog.

[34] Caragata EP, Rancès E, Hedges LM, Gofton AW, Johnson KN, O’Neill SL (2013). Dietary cholesterol modulates pathogen blocking by Wolbachia. PLoS Pathog.

[35] Frentiu FD (2017). Lipids and pathogen blocking by Wolbachia. Trends Parasitol.

[36] Geoghegan V, Stainton K, Rainey SM, Ant TH, Dowle AA, Larson T (2017). Perturbed cholesterol and vesicular trafficking associated with dengue blocking in Wolbachia-infected Aedes aegypti cells. Nat Commun.

[37] Osborne SE, Leong YS, O’Neill SL, Johnson KN (2009). Variation in antiviral protection mediated by different Wolbachia strains in Drosophila simulans. PLoS Pathog.

[38] Lu P, Bian G, Pan X, Xi Z (2012). Wolbachia induces density-dependent inhibition to dengue virus in mosquito cells. PLoS Negl Trop Dis.

[39] Ferguson NM, Hue Kien DT, Clapham H, Aguas R, Trung VT, Bich Chau TN, et al. Modeling the impact on virus transmission of Wolbachia-mediated blocking of dengue virus infection of Aedes aegypti. Sci Transl Med. 2015;7:279ra37. 10.1126/scitranslmed.3010370 PMC4390297 25787763

[40] Zink SD, van Slyke GA, Palumbo MJ, Kramer LD, Ciota AT (2015). Exposure to west nile virus increases bacterial diversity and immune gene expression in culex pipiens. Viruses.

[41] Hughes GL, Dodson BL, Johnson RM, Murdock CC, Tsujimoto H, Suzuki Y (2014). Native microbiome impedes vertical transmission of Wolbachia in Anopheles mosquitoes. Proc Natl Acad Sci U S A.

[42] Rossi P, Ricci I, Cappelli A, Damiani C, Ulissi U, Mancini MV (2015). Mutual exclusion of Asaia and Wolbachia in the reproductive organs of mosquito vectors. Parasit Vectors.

[43] Audsley MD, Seleznev A, Joubert DA, Woolfit M, O’Neill SL, McGraw EA (2017). Wolbachia infection alters the relative abundance of resident bacteria in adult Aedes aegypti mosquitoes, but not larvae. Mol Ecol.

[44] Wood DE, Lu J, Langmead B (2019). Improved metagenomic analysis with Kraken 2. Genome Biol.

[45] Matthews BJ, Dudchenko O, Kingan SB, Koren S, Antoshechkin I, Crawford JE (2018). Improved reference genome of Aedes aegypti informs arbovirus vector control. Nature.

[46] Morgulis A, Gertz EM, Schäffer AA, Agarwala R (2006). A fast and symmetric DUST implementation to mask low-complexity DNA sequences. J Comput Biol.

[47] Lu J, Breitwieser FP, Thielen P, Salzberg SL (2017). Bracken: estimating species abundance in metagenomics data. PeerJ Comput Sci.

[48] Team RC. R: a language and environment for statistical computing. 2021.

[49] Shannon CE (1948). A mathematical theory of communication. Bell Syst Tech J.

[50] Faust K, Raes J. CoNet app: inference of biological association networks using Cytoscape. F1000Research. 2016;5:1519. 10.12688/f1000research.9050.1 PMC5089131 27853510

[51] Shannon P, Markiel A, Ozier O, Baliga NS, Wang JT, Ramage D (2003). Cytoscape: a software environment for integrated models of biomolecular interaction networks. Genome Res.

[52] Oksanen J, Blanchet FG, Friendly M, Kindt R, Legendre P, McGlinn D, et al. vegan: community ecology package. 2020.

[53] Bray JR, Curtis JT (1957). An ordination of the upland forest communities of Southern Wisconsin. Ecol Monogr.

[54] Paradis E, Schliep K. ape 5.0: an environment for modern phylogenetics and evolutionary analyses in. Bioinformatics. 2019;35:526–8. 10.1093/bioinformatics/bty633 30016406

[55] Hegde S, Rasgon JL, Hughes GL (2015). The microbiome modulates arbovirus transmission in mosquitoes. Curr Opin Virol.

[56] Dickson LB, Ghozlane A, Volant S, Bouchier C, Ma L, Vega-Rúa A (2018). Diverse laboratory colonies of Aedes aegypti harbor the same adult midgut bacterial microbiome. Parasit Vectors.

[57] Osei-Poku J, Mbogo CM, Palmer WJ, Jiggins FM (2012). Deep sequencing reveals extensive variation in the gut microbiota of wild mosquitoes from Kenya. Mol Ecol.

[58] David MR, dos Santos LMB, Vicente ACP, Maciel-de-Freitas R (2016). Effects of environment, dietary regime and ageing on the dengue vector microbiota: evidence of a core microbiota throughout Aedes aegypti lifespan. Mem Inst Oswaldo Cruz.

[59] Arévalo-Cortés A, Mejia-Jaramillo AM, Granada Y, Coatsworth H, Lowenberger C, Triana-Chavez O (2020). The midgut microbiota of colombian aedes aegypti populations with different levels of resistance to the insecticide lambda-cyhalothrin. Insects.

[60] Yadav KK, Bora A, Datta S, Chandel K, Gogoi HK, Prasad GBKS (2015). Molecular characterization of midgut microbiota of Aedes albopictus and Aedes aegypti from Arunachal Pradesh India. Parasit Vectors.

[61] Ramirez JL, Souza-Neto J, Cosme RT, Rovira J, Ortiz A, Pascale JM (2012). Reciprocal tripartite interactions between the Aedes aegypti midgut microbiota, innate immune system and dengue virus influences vector competence. PLoS Negl Trop Dis.

[62] Gusmão DS, Santos A V., Marini DC, Bacci M, Berbert-Molina MA, Lemos FJA. Culture-dependent and culture-independent characterization of microorganisms associated with Aedes aegypti (Diptera: Culicidae) (L.) and dynamics of bacterial colonization in the midgut. Acta Trop. 2010;115:275–81. 10.1016/j.actatropica.2010.04.011 20434424

[63] Apte-Deshpande A, Paingankar M, Gokhale MD, Deobagkar DN. Serratia odorifera a midgut inhabitant of Aedes aegypti mosquito enhances its susceptibility to dengue-2 virus. PLoS One. 2012;7. 10.1371/journal.pone.0040401 PMC3407224 22848375

[64] Goto S, Anbutsu H, Fukatsu T (2006). Asymmetrical interactions between Wolbachia and Spiroplasma endosymbionts coexisting in the same insect host. Appl Environ Microbiol.

[65] Audsley MD, Ye YH, McGraw EA (2017). The microbiome composition of Aedes aegypti is not critical for Wolbachia-mediated inhibition of dengue virus. PLoS Negl Trop Dis.

[66] Gusmão DS, Santos AV, Marini DC, Russo ÉDS, Peixoto AMD, Bacci M (2007). First isolation of microorganisms from the gut diverticulum of Aedes aegypti (Diptera: Culicidae): new perspectives for an insect-bacteria association. Mem Inst Oswaldo Cruz.

[67] Yadav KK, Datta S, Naglot A, Bora A, Hmuaka V, Bhagyawant S, et al. Diversity of cultivable midgut microbiota at different stages of the Asian Tiger Mosquito, Aedes albopictus from Tezpur, India. PLoS One. 2016;11. 10.1371/journal.pone.0167409 PMC5152811 27941985

[68] Terenius O, Lindh JM, Eriksson-Gonzales K, Bussière L, Laugen AT, Bergquist H (2012). Midgut bacterial dynamics in Aedes aegypti. FEMS Microbiol Ecol.

[69] Ramos-Nino ME, Fitzpatrick DM, Eckstrom KM, Tighe S, Hattaway LM, Hsueh AN (2020). Metagenomic analysis of Aedes aegypti and Culex quinquefasciatus mosquitoes from Grenada West Indies. PLoS One.

[70] Scolari F, Casiraghi M, Bonizzoni M. Aedes spp. and their microbiota: a review. Front Microbiol. 2019;10. 10.3389/fmicb.2019.02036 PMC6738348 31551973

[71] Hegde S, Khanipov K, Albayrak L, Golovko G, Pimenova M, Saldaña MA (2018). Microbiome interaction networks and community structure from laboratory-reared and field-collected Aedes aegypti, Aedes albopictus, and culex quinquefasciatus mosquito vectors. Front Microbiol.

[72] Mejia AJ, Dutra HLC, Jones MJ, Perera R, McGraw EA (2022). Cross-tissue and generation predictability of relative Wolbachia densities in the mosquito Aedes aegypti. Parasit Vectors.

[73] Hoffmann AA, Iturbe-Ormaetxe I, Callahan AG, Phillips BL, Billington K, Axford JK (2014). Stability of the wMel Wolbachia Infection following Invasion into Aedes aegypti populations. PLoS Negl Trop Dis.

[74] Shade A, Handelsman J (2012). Beyond the Venn diagram: the hunt for a core microbiome. Environ Microbiol.

[75] Coon KL, Vogel KJ, Brown MR, Strand MR (2014). Mosquitoes rely on their gut microbiota for development. Mol Ecol.

[76] MacLeod HJ, Dimopoulos G, Short SM (2021). Larval diet abundance influences size and composition of the midgut microbiota of Aedes aegypti mosquitoes. Front Microbiol.

[77] Risely A (2020). Applying the core microbiome to understand host–microbe systems. J Anim Ecol.

[78] Yadav KK, Datta S, Naglot A, Bora A, Hmuaka V, Bhagyawant S (2016). Diversity of cultivable midgut microbiota at different stages of the Asian tiger mosquito, Aedes albopictus from Tezpur. India PLoS One.

[79] Osborne SE, Iturbe-Ormaetxe I, Brownlie JC, O’Neill SL, Johnson KN (2012). Antiviral protection and the importance of Wolbachia density and: tissue tropism in Drosophila simulans. Appl Environ Microbiol.

[80] Gupta V, Vasanthakrishnan RB, Siva-Jothy J, Monteith KM, Brown SP, Vale PF. The route of infection determines Wolbachia antibacterial protection in Drosophila. Proc R Soc B Biol Sci. 2017;284. 10.1098/rspb.2017.0809 PMC5474083 28592678

[81] Ye YH, Seleznev A, Flores HA, Woolfit M, McGraw EA (2017). Gut microbiota in Drosophila melanogaster interacts with Wolbachia but does not contribute to Wolbachia-mediated antiviral protection. J Invertebr Pathol.

[82] Ourry M, Crosland A, Lopez V, Derocles SAP, Mougel C, Cortesero AM, et al. Influential insider: Wolbachia, an intracellular symbiont, manipulates bacterial diversity in its insect host. Microorganisms. 2021;9. 10.3390/microorganisms9061313 PMC8234596 34208681

[83] Hooper L V., Littman DR, Macpherson AJ. Interactions between the microbiota and the immune system. Science (80). 2012;336:1268–73. 10.1126/science.1223490 PMC4420145 22674334

