# Supplementary material for: Aedes aegypti microbiome composition covaries with the density of Wolbachia infection
Source: Microbiome. 2023 Nov 17;11:255. doi: 10.1186/s40168-023-01678-9 (PMC10655336; doi:10.1186/s40168-023-01678-9)
Supplement: Supplementary file 7 — Additional file 6. Figure S5. PCoA of microbiome variation indicating collection site and historic release status. PCoA results including wMel are shown in Panel A and B. Individuals from sites with historic releases almost exclusively have positive loadings on Axis 1and negative loadings on Axis 3. PCoA results excluding wMel are shown in Panel C and D. Limited clustering by collection site or historic release status was observed. [file 40168_2023_1678_MOESM6_ESM.pdf]

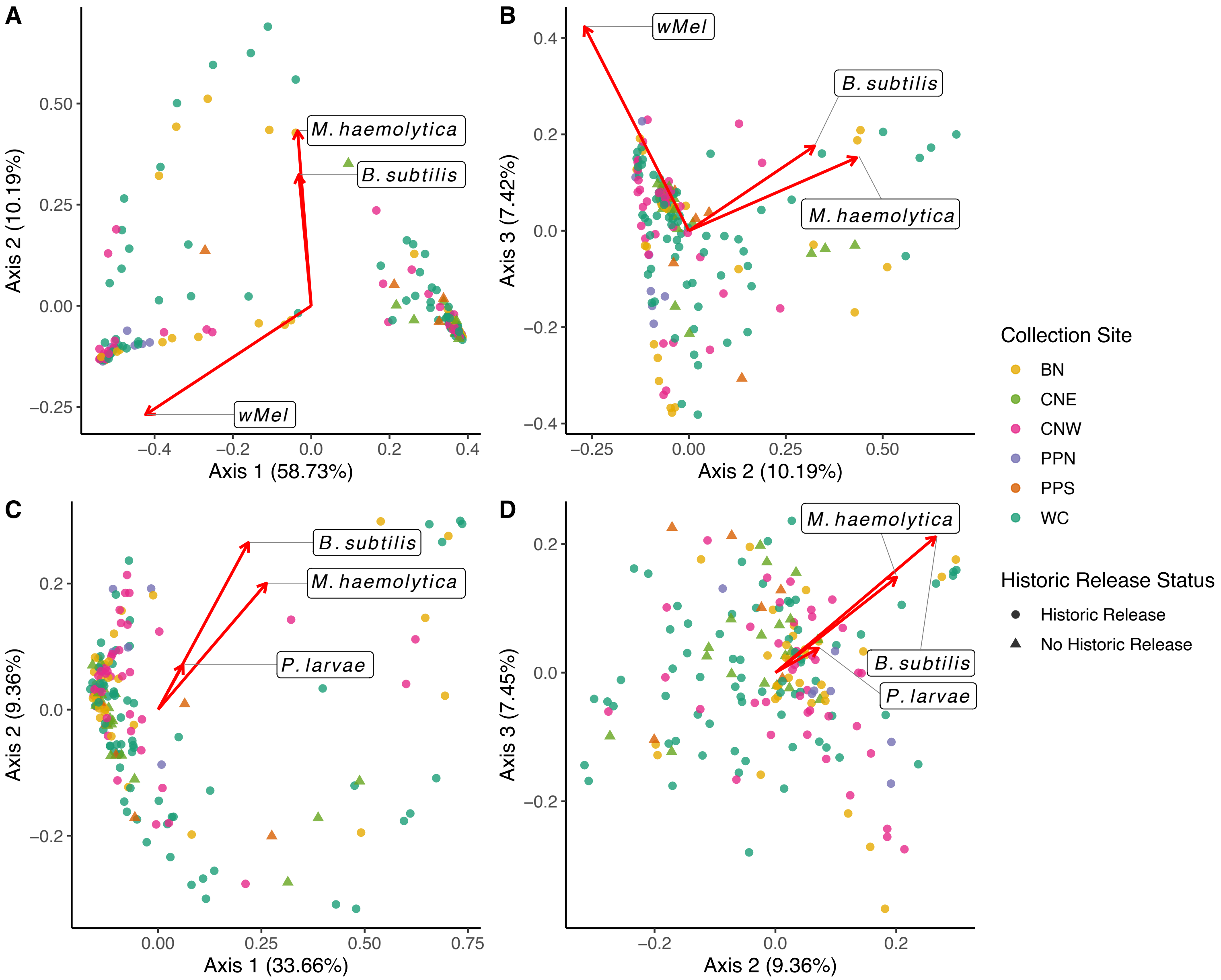

Figure S5. PCoA of microbiome variation indicating collection site and historic release status. PCoA results including *w*Mel are shown in Panel A and B. Individuals from sites with historic releases almost exclusively have positive loadings on PC #1 and negative loadings on PC #3. PCoA results excluding *w*Mel are shown in Panel C and D. Limited clustering by collection site or historic release status was observed.
